# Examining the association between sleep apnea and total hippocampal volumes in cognitive impairment

**DOI:** 10.1002/alz.70183

**Published:** 2025-04-25

**Authors:** Matthew Settimi, Dennis Tchoudnovski, Ivan Ntale, David R. Colelli, Yakdehikandage S. Costa, Sara Mitchell, Mario Masellis, Benjamin Lam, Andrew Lim, Frances Chung, Joel Ramirez, Maged Goubran, Sandra E. Black, Mark I. Boulos

**Affiliations:** ^1^ Hurvitz Brain Sciences Research Program Sunnybrook Research Institute Sunnybrook Health Sciences Centre, Wellness Way Toronto Ontario Canada; ^2^ Department of Medicine Division of Neurology University of Toronto Toronto Ontario Canada; ^3^ Undergraduate MD Program McMaster University Hamilton Ontario Canada; ^4^ Undergraduate MD Program 27 King's College Cir University of Toronto Toronto Ontario Canada; ^5^ UCD School of Medicine Health Sciences Centre University College Dublin, Dublin Belfield Ireland; ^6^ Undergraduate MD Program Memorial University, Newfoundland and Labrador St. John's Canada; ^7^ Department of Psychiatry University of Toronto Toronto Ontario Canada; ^8^ Sunnybrook Sleep Laboratory North York Ontario Canada; ^9^ Department of Anesthesia and Pain Management Toronto Western Hospital University Health Network University of Toronto Toronto Ontario Canada; ^10^ Dr. Sandra Black Centre for Brain Resilience and Recovery Sunnybrook Research Institute Sunnybrook Health Sciences Centre North York Ontario Canada; ^11^ Physical Sciences Platform Sunnybrook Research Institute Sunnybrook Health Sciences Centre North York Ontario Canada; ^12^ Department of Medical Biophysics University of Toronto Toronto Ontario Canada

**Keywords:** AHI, Apnea Hypopnea Index, brain imaging, hippocampal volume, home sleep apnea test, HSAT, neurodegenerative disorders, neuroimaging, obstructive sleep apnea, OSA, polysomnography, vascular dementia

## Abstract

**INTRODUCTION:**

The prevalence of dementia is rising due to an aging population. Given the known risks of obstructive sleep apnea (OSA) on cerebrovascular health, we hypothesized that markers of OSA would correlate with reduced hippocampal volumes in individuals with cognitive impairment due to neurodegenerative, vascular, or mixed (NVM) etiologies.

**METHODS:**

Data from 166 patients were retrospectively analyzed. Participants underwent polysomnography or home sleep apnea tests, alongside structural brain magnetic resonance imaging (MRI). They were categorized into cognitive impairment due to NVM etiology or subjective cognitive complaints. Multiple linear regression models examined correlations between sleep variables and hippocampal volume.

**RESULTS:**

The presence of OSA, time under 90% oxygen saturation, and mean SpO_2_ were significantly associated with reduced hippocampal volumes in the NVM group, but not the subjective cognitive complaints.

**DISCUSSION:**

These findings suggest that individuals with cognitive impairment due to NVM etiology experience underlying neurodegenerative processes, which increase susceptibility to the adverse effects of OSA.

**Highlights:**

- Hypoxic burden, rather than AHI, was associated with hippocampal volume loss.
- The presence of OSA, T90%, and mean SpO_2_ were linked with reduced hippocampal volume.
- Neurodegenerative and vascular processes may raise susceptibility to OSA harms.

## 1 BACKGROUND

Cognitive impairment and dementia are becoming increasingly pervasive and burdensome in an aging demographic. A World Health Organization report estimated that in 2019, there were 59 million patients worldwide living with dementia, a number that is expected to triple by 2050. Dementia is also financially burdensome to both patients and caregivers, with worldwide costs expected to double from US$1.3 trillion per year in 2019 to US$2.8 trillion by 2030.^1^, ^2^ Age‐related cognitive impairment can manifest itself through several different etiological and pathophysiological mechanisms, with most causes typically being neurodegenerative (e.g., Alzheimer's, Parkinsons’ dementia), vascular (e.g., vascular dementia), or mixed in nature. Given the heterogeneity of these conditions and the increasing burdens associated with them, there is great interest in developing novel therapeutic targets for patients.

Obstructive sleep apnea (OSA), characterized by the repetitive partial or complete interruption of breathing during sleep, causes fragmented sleep and intermittent hypoxemia.^3^, ^4^ This results in sympathetic activation with blood pressure elevation, oxidative stress, and vascular inflammation.^5^ Over time, these harmful physiological changes increase the risk of developing cerebrovascular events and cognitive impairment. Specifically, studies have shown that patients with OSA and other forms of sleep‐disordered breathing are twice as likely to suffer from a fatal or non‐fatal stroke and 26% more likely to develop cognitive impairment.^6^, ^7^, ^8^

To better understand the cognitive effects of OSA, neuroimaging studies often focus on the hippocampus, an area of the brain central to cognitive ability and known to be sensitive to hypoxic damage.^9^, ^10^, ^11^, ^12^ Prior studies showed that OSA was associated with decreased bilateral hippocampal gray matter and decreased total and right hippocampus volumes.^13^, ^14^ Similarly, another study demonstrated that treatment of OSA with continuous positive airway pressure (CPAP) was associated with increased hippocampal gray matter volume.^15^ Notably, prior studies investigating the link between OSA and hippocampal measures only used cohorts of patients with normal cognition or lacking stratification by cognition.

The aim of this study was to determine whether sleep apnea was associated with reduced hippocampal volumes in individuals with cognitive impairment due to a neurodegenerative, vascular, or mixed (NVM) etiology compared to individuals with subjective cognitive complaints. We hypothesized that the presence and severity of OSA would be negatively associated with hippocampal volumes in patients with cognitive impairment of NVM etiology but not in those with subjective cognitive complaints.

## 2 METHODS

### 2.1 Study design and population

Patient data from Sunnybrook Health Sciences Centre were retrospectively reviewed. All participants were patients who had visited the cognitive neurology clinic at Sunnybrook Health Sciences Centre between 2009 and 2021 and were retrieved from one of the following three sources: a feasibility study examining the unattended use of home sleep apnea tests (HSAT) in an impaired population,^16^ the ENhancing Outcomes in Cognitive Impairment Through Use of Home Sleep ApNea Testing (ENCHANT) study,^17^ or a retrospective chart review of all patient visits at the Sunnybrook Health Sciences Centre sleep laboratory,^18^ from 2009 through to the end of 2021. All participants in these studies completed a sleep study (in‐laboratory polysomnography [PSG] or HSAT), in addition to a structural magnetic resonance imaging (MRI) brain.

### 2.2 Eligibility criteria

Inclusion criteria for participation in this retrospective analysis included:

1. Diagnosed with cognitive impairment by a cognitive neurologist at the Sunnybrook Health Sciences Centre cognitive neurology clinic (an academic tertiary care clinic for dementia) due to a NVM etiology (diagnoses included Alzheimer's disease, mild cognitive impairment,^19^, ^20^ vascular cognitive impairment and dementia,^21^ dementia with Lewy bodies, Parkinson's disease dementia,^22^, ^23^ and/or mixed disease, using standardized criteria) or those with subjective cognitive complaints as diagnosed by a cognitive neurologist for patients with self‐reported cognitive decline but not meeting criteria for a neurodegenerative and/or vascular etiology. Notably, those with subjective cognitive complaints may have underlying neurodegenerative and/or vascular concerns; however, they did not meet diagnostic criteria at the time of testing and may have progressed after the time of our data collection. For further disambiguation of diagnostic criteria, please refer to Table S1 in the Supporting Information. Specific cognitive testing scores were not available in the dataset.
2. Underwent a sleep study and acquired usable sleep data, either via in‐laboratory PSG or HSAT.^16^ Sleep data were judged to be usable if ≥4 h of flow, effort, and oxygen evaluation were obtained.^16^, ^24^
3. The acquisition of usable structural MRI data within 12 months (before or after) of either PSG or HSAT recording. MRI data were judged to be usable if they were free of motion and other imaging artifacts and contained the following necessary sequences for image processing: three‐dimensional T1‐weighted (3DT1) and T2‐weighted fluid‐attenuated inversion recovery (T2‐FLAIR).

Exclusion criteria for participation included:

1. Diagnosis of cognitive impairment not of neurodegenerative and/or vascular origin (such as impairments caused by traumatic brain injury or post‐concussion syndrome).
2. Absence of subjective cognitive complaints diagnosis.
3. Moderate to severe pulmonary disease or congestive heart failure were excluded as those can compromise the validity of HSAT results.

**RESEARCH IN CONTEXT**

1. **Systematic review**: The authors reviewed the literature using traditional sources to evaluate primary and secondary research articles, as well as relevant presentations. The relationships between sleep‐disordered breathing and different measures of brain volumetrics have been reported on, and we cite relevant past research here.
2. **Interpretation**: Our findings provide evidence for the hypothesis that cognitive impairment due to a NVM etiology causes changes that increase susceptibility to the adverse effects of OSA as seen by a decrease in hippocampal volume.
3. **Future directions**: This research provides a foundation for further research into the role that sleep‐disordered breathing plays in brain health and cognition. Future research in this area can explore how treating OSA may affect cognitive decline in individuals with various forms of cognitive impairment such as Alzheimer's disease, mild cognitive impairment, vascular cognitive impairment, and Parkinson's disease.

### 2.3 Neuroimaging and sleep study data

Participants who underwent HSAT did so using the ApneaLink Air,^25^ which has been validated against PSG for the detection of OSA.^16^, ^26^ The ApneaLink Air measures three channels: oxygen saturation via a pulse oximeter, movements associated with respiration via a chest effort sensor, and airflow via a nasal cannula. HSAT recordings were analyzed automatically through the ApneaLink software and were later manually scored by a sleep physician in accordance with the American Academy of Sleep Medicine guidelines.^27^

The remaining participants underwent level 1 in‐laboratory PSG at the Sunnybrook Sleep Laboratory. These overnight PSGs were monitored and scored by a sleep technologist in accordance with the guidelines set by the American Academy of Sleep Medicine.^27^ For the purposes of our analysis, the presence of OSA was defined as an Apnea Hypopnea Index (AHI) greater than or equal to 15 or an AHI greater than or equal to 5 and less than 15 events per hour with the lowest oxygen desaturation (SpO_2_) of 88% or lower.^28^ Given that the present literature suggests a major overlap between cognitively impaired patients and stroke patients, this definition of OSA, as utilized by Patel et al., was chosen.^21^, ^28^ The AHI was defined as the number of apnea and hypopnea events per hour of sleep; apnea events were defined as a ≥90% reduction in airflow for ≥10 s, while hypopnea events were defined as a ≥30% reduction in airflow for ≥10 s, with an associated ≥4% oxygen desaturation.^27^ Time under 90% oxygen saturation (T90%) was defined as the proportion of cumulative sleep time spent with a SpO_2_ below 90%. Mean SpO_2_ was defined as the mean oxygen saturation levels for the duration of sleep. Together, the AHI, T90%, and mean SpO_2_ were used as metrics of OSA severity.

All study participants underwent a structural brain MRI at Sunnybrook Health Sciences Centre. 3DT1 and T2‐FLAIR scans were acquired using either a 1.5 Tesla Signa system,^29^ a 3 Tesla Discovery MR750 system,^30^ or a 3 Tesla Prisma system.^31^ Structural brain MRI scans were processed to measure hippocampal and intracranial volumes using the HippMapp3r and iCVMapper pipelines, respectively.^32^, ^33^

### 2.4 Statistical analysis

Descriptive statistics were generated for this study's participant pool. Specifically, frequency counts were performed for categorical variables, while mean and standard deviations were calculated for continuous variables. These data were analyzed to explore significant differences between participants with diagnosed cognitive impairment of NVM etiology and those with subjective cognitive complaints, as well as between those with and without OSA. Independent *t*‐tests, chi‐squared tests for independence, and Fisher's exact tests were used for these comparisons where appropriate.

Volumetrics from the structural brain MRI data and sleep study parameters were investigated using multiple linear regression and interaction analyses. For each relationship explored, a minimally adjusted and fully adjusted model were run. Minimally adjusted models included age, sex, and body mass index (BMI) as covariates. The fully adjusted models included the covariates from the minimally adjusted models, as well as intracranial volume, years of education, and history of hypertension, diabetes, and/or stroke. Covariates were selected in keeping with neuroimaging study practices under the justification that hippocampal volume is likely positively associated with intracranial volume and increased educational attainment.^34^, ^35^ Additionally, hypertension, diabetes, and stroke are associated with cerebrovascular disease and hippocampal integrity. All statistics described above were computed using R version 3.5.1 on a Windows 10 workstation.

### 2.5 Objectives

Our primary objective was to determine whether the presence of OSA was associated with decreased total hippocampal volume in patients diagnosed with cognitive impairment due to NVM etiology. The secondary objective was to determine whether OSA severity was associated with total hippocampal volume in patients with cognitive impairments due to NVM etiology. To quantify OSA severity, three different sleep metrics were used: (a) AHI, (b) T90%, and (c) mean SpO_2_. Both objectives were additionally compared to a cohort of individuals diagnosed with subjective cognitive complaints, who served as controls, and were predicted to not show changes in their hippocampal volume.

An additional analysis was also performed, examining the effects of the sleep variables on left and right hippocampal volumes individually. Additionally, a subgroup analysis was performed examining the differences between specific etiology types in individuals with cognitive impairment due to NVM etiology (i.e., individually examining NVM etiologies).

## 3 RESULTS

### 3.1 Patient flow and characteristics

**FIGURE 1:**
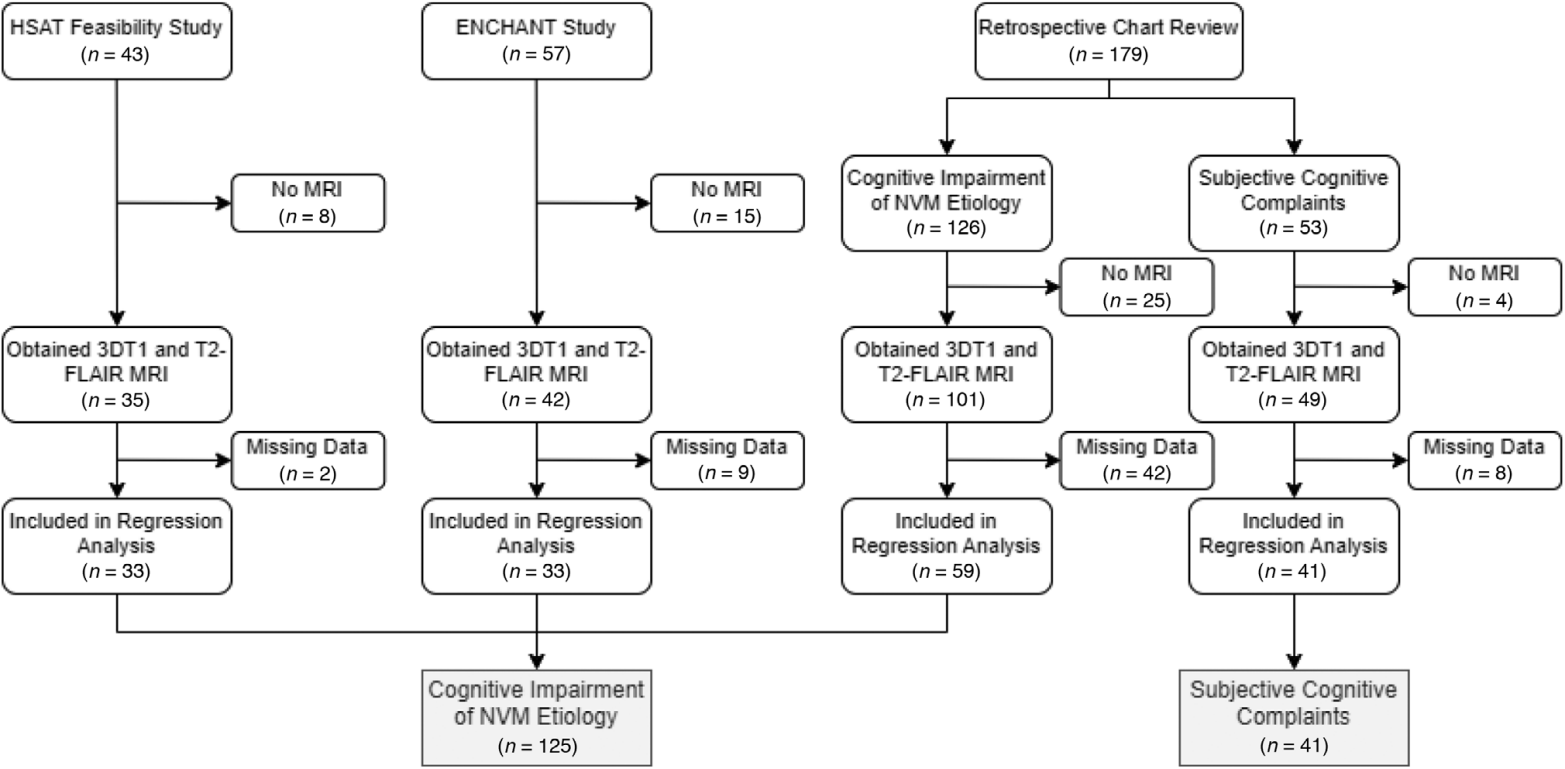
Flow chart illustrating progression of participants from three distinct sources through study to final analyzed cohort. Exclusions due to absence of MRI data and other missing data are indicated. MRI, magnetic resonance imaging.

A review was conducted on 279 patients who had undergone a sleep study via a HSAT or at the Sunnybrook Sleep Laboratory from February 2009 to August 2022. Among these, 43 patients originated from a feasibility study for unattended HSAT testing in a cognitively impaired population,^16^ 57 from the ENCHANT study,^17^ and 179 from a retrospective chart review.^18^ After the inclusion of only those who received a 3DT1 and T2‐FLAIR MRI and the exclusion of those with missing data, 166 patients remained. Of these patients, 41 had subjective cognitive complaints and served as controls, whereas the remaining 125 were diagnosed with cognitive impairment secondary to a NVM etiology. Figure 1 summarizes the recruitment strategy and flow of patients.

Demographic data are summarized in Table 1, stratified individually by cognitive status and OSA presence. Individuals with cognitive impairment due to a NVM etiology had an overall greater age than those with subjective cognitive complaints. Similarly, when stratifying by OSA, it was noted that those with OSA had an overall greater mean age. The proportion of males was greater in both the cognitive impairment due to NVM etiology group (57%) and the OSA group (64%) compared to their respective counterparts. Table 2 demonstrates demographic data of those with cognitive impairment due to NVM etiology, stratified by origin (NVM). Notably, the majority of patients diagnosed with cognitive impairment were of neurodegenerative etiology. Table 3 summarizes demographic data, stratified by cognitive status in the presence or absence of OSA. Notably, a significant difference in total hippocampal volume was noted between those with and without OSA, specifically in patients with cognitive impairment of NVM etiology. Those with subjective cognitive complaints did not demonstrate a statistically significant difference in their total hippocampal volume regardless of their OSA status.

**TABLE 1:** Demographic information stratified by type of cognitive impairment and OSA.

Characteristic	Cognitive impairment of NVM etiology (*N* = 125)	Subjective cognitive complaints (*N* = 41)	OSA (*N* = 84)	No OSA (*N* = 82)
Age in years, mean (SD)	**68.7 (11.1)**	**54.2 (11.6)**	**69.5 (10.2)**	**60.7 (13.8)**
Sex – male, % (*N*)	**57% (71)**	**37% (15)**	**64% (54)**	**39% (32)**
BMI, mean (SD)	26.5 (4.5)	27.0 (4.4)	27.1 (4.7)	26.2 (4.1)
Intracranial volume in mm^3^, mean (SD)	1,402,329 (132,263)	1,385,183 (136,382)	1,411,896 (123,761)	1,383,956 (141,359)
Total hippocampal volume in mm^3^, mean (SD)	**5,927 (1019)**	**6,892 (843)**	**5,853 (977)**	**6,484 (1056)**
Years of education, mean (SD)	**15.0 (3.9)**	**17.0 (2.5)**	15.4 (3.6)	15.7 (3.8)
Hypertension, % (*N*)				
None	**43% (54)**	**76% (31)**	44% (37)	59% (48)
Treated	**53% (66)**	**22% (9)**	50% (42)	40% (33)
Untreated	**4% (5)**	**2% (1)**	6% (5)	1% (1)
Diabetes, % (*N*)				
None	**81% (102)**	**98% (40)**	85% (71)	87% (71)
Treated	**18% (22)**	**2% (1)**	15% (13)	12% (10)
Untreated	**1% (1)**	**0% (0)**	0% (0)	1% (1)
History of stroke, % (*N*)	14% (17)	10% (4)	12% (10)	13% (11)
OSA, % (*N*)	**57% (71)**	**32% (13)**	–	–
AHI, mean (SD)	**10.5 (12.7)**	**6.3 (8.7)**	**16.7 (13.0)**	**2.0 (2.1)**
T90%, mean (SD)	**7.4 (13.8)**	**1.2 (3.4)**	**9.6 (15.8)**	**2.0 (5.3)**
Mean SpO_2_, mean (SD)	**93.8 (2.0)**	**95.0 (1.6)**	**93.2 (1.7)**	**95.1 (1.8)**
Cognitive impairment of NVM etiology, % (N)	–	–	**85% (71)**	**66% (54)**

*Note*: Bolded values indicate statistical difference.

Abbreviations: AHI, Apnea Hypopnea Index; NVM, neurodegenerative, vascular, or mixed; OSA, obstructive sleep apnea; SD, standard deviation; T90%, time under 90% oxygen saturation.

**TABLE 2:** Demographic information of individuals with cognitive impairment of neurodegenerative, vascular, or mixed etiology stratified by type.

Characteristic	Neurodegenerative (*N* = 92)	Vascular (*N* = 21)	Mixed (*N* = 12)
Age in years, mean (SD)	68.6 (10.3)	68.3 (14.7)	70.2 (11.5)
Sex – male, % (*N*)	59% (54)	57% (12)	42% (5)
BMI, mean (SD)	26.4 (4.1)	26.5 (4.6)	27.5 (6.6)
Intracranial volume in mm^3^, mean (SD)	1,417,329 (131,974)	1,370,101 (131,995)	1,343,731 (117,581)
Total hippocampal volume in mm^3^, mean (SD)	5939 (1081)	6123 (664)	5485 (980)
Years of education, mean (SD)	15.0 (4.1)	14.9 (3.3)	15.8 (3.5)
Hypertension, % (*N*)			
None	42% (39)	43% (9)	50% (6)
Treated	54% (50)	48% (10)	50% (6)
Untreated	3% (3)	10% (2)	0% (0)
Diabetes, % (*N*)			
None	86% (79)	71% (15)	67% (8)
Treated	13% (12)	29% (6)	33% (4)
Untreated	1% (1)	0% (0)	0% (0)
History of stroke, % (*N*)	96% (88)	52% (11)	75% (9)
OSA, % (*N*)	52% (48)	57% (12)	92% (11)
AHI, mean (SD)	9.8 (12.1)	12.3 (17.6)	11.9 (6.1)
T90%, mean (SD)	7.6 (13.9)	4.4 (8.4)	11.5 (19.8)
Mean SpO_2_, mean (SD)	93.9 (2.1)	93.9 (1.9)	93.2 (1.9)

Abbreviations: AHI, Apnea Hypopnea Index; BMI, body mass index; OSA, obstructive sleep apnea; SD, standard deviation; T90%, time under 90% oxygen saturation.

**TABLE 3:** Demographic information for cognitive impairment grouped by presence or absence of obstructive sleep apnea.

Characteristic	NVM OSA− (*N* = 54)	NVM OSA+ (*N* = 71)	SCC OSA− (*N* = 28)	SCC OSA+ (*N* = 13)
Age in years, mean (SD)	**65.8 (11.9)**	**71.0 (10.0)**	**50.9 (11.9)**	**61.3 (7.0)**
Sex – male, % (*N*)	**39% (21)**	**70% (50)**	39% (11)	31% (4)
BMI, mean (SD)	25.8 (4.0)	27.0 (4.7)	26.9 (4.3)	27.3 (4.8)
Intracranial volume in mm^3^, mean (SD)	**1,374,830 (138,990)**	**1,423,244 (123,834)**	1,401,556 (146,761)	1,349,918 (107,541)
Total hippocampal volume in mm^3^, mean (SD)	**6227 (1037)**	**5698 (950)**	6979 (921)	6704 (638)
Years of education, mean (SD)	15.0 (4.1)	15.1 (3.7)	17.1 (2.6)	16.8 (2.4)
Hypertension, % (*N*)				
None	46% (25)	41% (29)	82% (230	62% (8)
Treated	52% (28)	54% (38)	18% (5)	31% (4)
Untreated	2% (1)	6% (4)	0% (0)	8% (1)
Diabetes, % (*N*)				
None	80% (43)	83% (59)	100% (28)	62% (8)
Treated	19% (10)	17% (12)	0% (0)	31% (4)
Untreated	2% (1)	0% (0)	0% (0)	8% (1)
History of stroke, % (*N*)	17% (9)	11% (8)	7% (2)	15% (2)
AHI, mean (SD)	**2.0 (2.3)**	**16.9 (13.6)**	**1.8 (1.8)**	**16.0 (9.8)**
T90%, mean (SD)	**2.7 (6.0)**	**11.0 (16.8)**	0.8 (3.5)	2.1 (2.8)
Mean SpO_2_, mean (SD)	**95.0 (1.9)**	**93.0 (1.7)**	**95.4 (1.6)**	**94.2 (1.5)**

*Note*: Bolded values indicate statistical difference.

Abbreviations: AHI, Apnea Hypopnea Index; NVM, neurodegenerative, vascular, or mixed; OSA, obstructive sleep apnea; SCC, Subjective Cognitive Complaints; SD, standard deviation; T90%, time under 90% oxygen saturation.

### 3.2 Association between OSA and total hippocampal volume

For patients with cognitive impairment due to a NVM etiology, our minimally adjusted model, which controlled for age, sex, and BMI, demonstrated a significantly lower total hippocampal volume in patients with OSA (β: −536.3 [95% CI: −875.7 to −196.9]; *p* = 0.002) compared to those without OSA. Similarly, with the inclusion of additional variables (intracranial volume, years of education, hypertension, diabetes, and stroke) in the fully adjusted model, OSA was also associated with decreased total hippocampal volume (β: −573.4 [95% CI: −891.2 to −255.6]; *p* = 0.001). Conversely, in the subjective cognitive complaints group, both the minimally and fully adjusted models did not reach statistical significance (*p* = 0.891 and *p* = 0.855, respectively). In the combined model, which included all participants regardless of cognitive status, the interaction of cognitive impairment due to NVM etiology with OSA was examined; both variables were assessed as binary variables and the interaction of these two was associated with significant reductions in total hippocampal volume (*β*: −836.6 [95% CI: −1422.0 to −251.2]; *p* = 0.002).

### 3.3 Association between AHI and total hippocampal volume

AHI, utilized as a measure of OSA severity, was not shown to be associated with decreased total hippocampal volume in any of the groups. Specifically, in the cognitive impairment by a NVM etiology group, the minimally and fully adjusted models did not demonstrate significance (*p* = 0.188 and *p* = 0.111, respectively). Similarly, in the subjective cognitive complaints group, neither reached statistical significance (*p* = 0.824 and *p* = 0.456, respectively).

### 3.4 Association between T90% and total hippocampal volume

T90% was also used as a measure of OSA severity. In the cognitive impairment by a NVM etiology group, T90% was associated with a statistically significant reduction in total hippocampal volume in both the minimally (β: −11.5 [95% CI: −23.0 to −0.0]; *p* = 0.050) and fully adjusted models (β: −11.8 [95% CI: −22.5 to −1.0]; *p* = 0.032). Conversely, neither model in the subjective cognitive complaints group was statistically significant (*p* = 0.0954 in the minimally adjusted model; *p* = 0.882 in the fully adjusted model).

### 3.5 Associations between mean SpO_2_ and total hippocampal volume

Mean SpO_2_, another metric of OSA severity, was also shown to be associated with decreased hippocampal volumes in patients with cognitive impairment due to NVM etiology. Statistical significance was demonstrated in both the minimally adjusted model (β: 92.3 [95% CI: 10.6 to 173.9]; *p* = 0.027) and the fully adjusted model (β: 118.3 [95% CI: 43.7 to 193.0]; *p* = 0.002). Alternatively, in the subjective cognitive complaints group, statistical significance was not reached (*p* = 0.262 in the minimally adjusted model; *p* = 0.375 in the fully adjusted model).

### 3.6 Bivariate analysis of sleep variables and total hippocampal volumes

**FIGURE 2:**
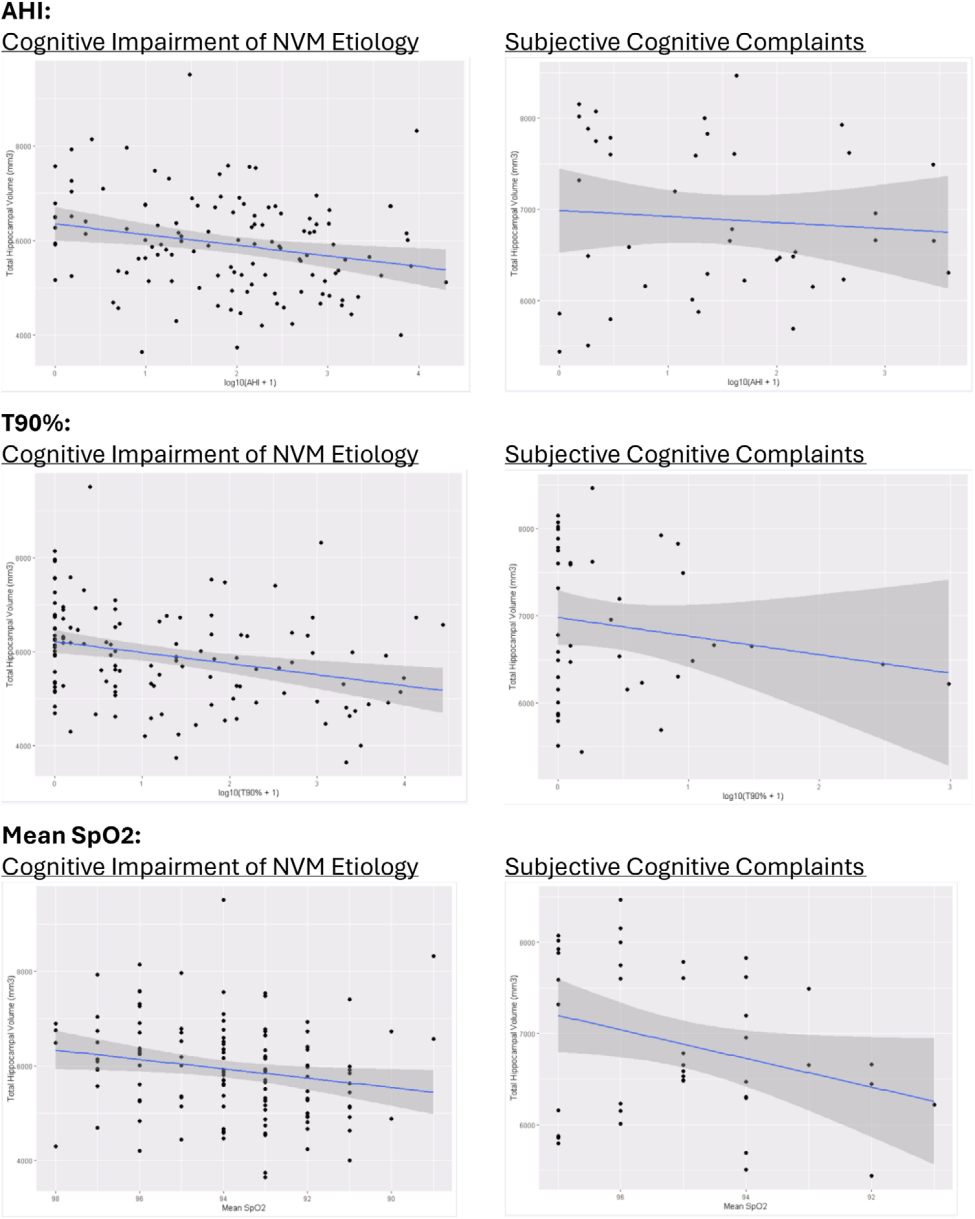
Scatterplots examining the relationship between hippocampal volume and sleep variables (AHI, T90%, and mean SpO_2_), stratified by cognitive status. Stratified groups include participants with cognitive impairment due to NVM etiology and those with subjective cognitive complaints. AHI, Apnea Hypopnea Index; NVM, neurodegenerative, vascular, or mixed.

Figure 2 displays the relationship between the analyzed sleep variables and total hippocampal volumes. Although the associations are not as statistically robust as those observed in the regression analyses, a general downward trend is apparent across the variables. Notably, the subjective cognitive complaints group exhibits larger confidence intervals, indicating greater variability in this group.

### 3.7 Effects of sleep variables on left and right hippocampal volumes

To assess whether hippocampal atrophy occurred preferentially on one side, fully adjusted regression models were run, examining each hemisphere individually. When examining the left and right hippocampal volumes individually, similar results were obtained as compared to the total hippocampal volume analyses. Specifically, the presence of OSA and mean SpO_2_ (but not AHI) were associated with decreased left and right hippocampal volumes in individuals with cognitive impairment due to NVM etiology. There was one exception as the T90%, while significantly associated with decreased left hippocampal volumes, did not reach statistical significance in its association for reduced right hippocampal volumes in individuals with cognitive impairment due to NVM etiology. As expected, within the subjective cognitive complaints group, no statistically significant relation was identified between the presence of OSA, AHI, T90%, and mean SpO_2_ with reduced hippocampal volumes on either side. Thus, for the subjective cognitive complaint, the analysis of left and right hippocampal volumes mimicked the analysis of total hippocampal volumes. Results are further disambiguated below in Tables 4 and 5.

**TABLE 4:** Associations between sleep variables and cognitive impairment due to neurodegenerative, vascular, or mixed etiology in reduction of left and right hippocampal volumes.

Sleep Parameter	Hippocampal region	β	95% CI	*P*
OSA	Left	−298.5	−456.1 to −141.0	**<0.001**
	Right	−274.9	−455.3 to −94.5	**0.003**
AHI	Left	−3.4	−9.5 to 2.8	0.283
	Right	−6.6	−13.5 to 0.2	0.058
T90%	Left	−6.2	−11.5 to −0.8	**0.024**
	Right	−5.6	−11.6 to 0.4	0.069
Mean SpO_2_	Left	57.1	19.9 to 94.4	**0.003**
	Right	61.2	19.2 to 103.2	**0.005**

*Notes*: This table demonstrates the results of running the fully adjusted model to test the four sleep variables (OSA, AHI, T90%, and mean SpO_2_) and their association with reduced hippocampal volumes, specifically stratified to the left and right lobes, in individuals with cognitive impairment due to NVM etiology. The fully adjusted model included the following variables: age, sex, body mass index (BMI), intracranial volume, years of education, and history of hypertension, diabetes, and/or stroke. Bolded values indicate P<0.05

Abbreviations: AHI, Apnea Hypopnea Index; CI, confidence interval; OSA, obstructive sleep apnea.

**TABLE 5:** Associations between sleep variables and subjective cognitive complaints in reduction of left and right hippocampal volumes.

Sleep Parameter	Hippocampal region	β	95% CI	*P*
OSA	Left	107.6	−208.0 to 423.1	0.492
	Right	−61.5	−349.2 to 226.2	0.665
AHI	Left	8.1	−10.7 to 26.9	0.386
	Right	3.1	−14.1 to 20.4	0.714
T90%	Left	9.0	−32.1 to 50.2	0.658
	Right	−4.1	−41.6 to 33.3	0.823
Mean SpO_2_	Left	33.3	−61.9 to 128.5	0.480
	Right	33.9	−52.3 to 120.1	0.428

*Notes*: This table presents the results of running the fully adjusted model to test the four sleep variables (OSA, AHI, T90%, and mean SpO_2_) and their association with reduced hippocampal volumes, specifically stratified to the left and right lobes, in individuals with subjective cognitive complaints. The fully adjusted model included the following variables: age, sex, body mass index (BMI), intracranial volume, years of education, and history of hypertension, diabetes, and/or stroke.

Abbreviations: AHI, Apnea Hypopnea Index; CI, confidence interval; OSA, obstructive sleep apnea.

### 3.8 Differences in etiology for NVM cognitive impairment

In individuals with cognitive impairment of NVM etiology, a subgroup analysis was performed to examine whether the presence of OSA, AHI, T90%, and mean SpO_2_ was associated with reduced hippocampal volumes – specifically within the three distinct etiology types (NVM). Linear regression modeling, incorporating the same factors as the fully adjusted model in the primary objective, was conducted for the neurodegenerative cohort due to its sufficient sample size and statistical power. In contrast, Spearman correlation analyses were used for the vascular and mixed groups.

Linear regression identified that OSA was predictive of decreased hippocampal volumes in individuals with neurodegenerative cognitive impairment (β: −588.6 [95% CI: −993.8 to −183.4]; *p* = 0.005). Conversely, correlation analyses did not demonstrate an associated decrease in hippocampal volumes for vascular (*p* = 0.681) and mixed (*p* = 0.6848) cognitive impairment in the presence of OSA.

When evaluating the association between AHI and reduced hippocampal volumes, none of the distinct etiologies of cognitive impairment showed statistically significant results (neurodegenerative: *p* = 0.163, vascular: *p* = 0.1641, mixed: *p* = 0.6834). Notably, T90% was not found to be associated with reduced hippocampal volume in those with neurodegenerative (*p* = 0.084), vascular (*p* = 0.05611), or mixed (*p* = 0.4849) etiology. The mean SpO_2_ was found to be associated with reduced hippocampal volume in those with neurodegenerative cognitive impairment (β: 116.3 [95% CI: 27.8 to 204.9]; *p* = 0.011). Correlation analyses were not significant for a vascular (*p* = 0.08386) or mixed (*p* = 0.5368) etiology.

## 4 DISCUSSION

This study of 166 participants explored the relationship between sleep apnea and hippocampal volume in a cohort of cognitively impaired individuals. Specifically, we examined several sleep‐related variables, including the presence of OSA, AHI, T90%, and mean SpO_2_, and their relation to the total hippocampal volume as measured by structural MRI. We demonstrated that the presence of OSA, T90%, and mean SpO_2_ was significantly associated with reduced hippocampal volumes in individuals with cognitive impairment due to NVM etiology, but not in those with subjective cognitive complaints.

The primary objective of this study was to explore the effects of OSA on hippocampal volume. In individuals with cognitive impairment due to NVM etiologies, OSA (defined by AHI ≥ 15 or AHI ≥ 5 to 15 with SpO_2_ ≤88%)^28^ was associated with reduced hippocampal volume, a relationship not observed in those with subjective cognitive complaints. Previous studies identified a link between OSA and hippocampal atrophy but were often composed of cohorts with normal cognition or already cognitively impaired individuals or lacked stratification into these subgroups.^36^, ^37^ A 2019 study by Owen et al. demonstrated this relationship through the examination of 32 patients with prior OSA diagnoses via autopsy and histopathology.^38^ They identified that OSA was associated with hippocampal volume loss, with severity correlating to greater atrophy. Notably, cognitive status was unassessed. Additionally, Marchi et al. reported that sleep‐apnea‐related hypoxemia was associated with lower fornix integrity in MCI subjects but not unimpaired individuals. Given the close connection between the hippocampus and fornix, these findings suggest that hypoxic susceptibility is more pronounced in patients with MCI, a form of neurodegenerative cognitive impairment.^39^ Our findings support this unique association as OSA's correlation with decreased hippocampal volumes was shown to be specific to individuals with cognitive impairment due to NVM etiology. This aligns with our hypothesis that patients with cognitive impairment of NVM etiology have underlying neurodegenerative processes that make them more vulnerable to the detrimental effects of OSA on hippocampal volume. This was further validated by a combined model analysis, which included both NVM cognitive impairment and subjective cognitive complaints as one cohort. The analysis demonstrated that alone, neither OSA nor cognitive impairment due to NVM etiology were associated with reduced hippocampal volumes. Rather, the interaction of these variables was significantly associated with atrophy. Table 3 additionally highlights how hippocampal volumes only significantly differed based on the presence of OSA within individuals with cognitive impairment due to NVM etiology. Evidently, it is the presence of OSA in those that are cognitively impaired due to a NVM etiology that likely increases susceptibility to hippocampal atrophy.^4^, ^9^, ^10^, ^11^, ^12^ Additionally, this supports the possibility that sleep apnea may be a component of various neurodegenerative disorders such as Alzheimer's disease and mixed dementia.

When assessing the association of the AHI with hippocampal volume, no associations were identified in either the cognitive impairment due to a NVM etiology or subjective cognitive complaints group. Our hypothesis is that this lack of correlation is due to the inaccuracy of the AHI as a measurement for the severity of OSA. Specifically, recent studies have demonstrated that the AHI alone, without the consideration of desaturation criteria, has not been shown to be predictive of functional impairment or decrease in quality of life associated with OSA.^4^, ^40^, ^41^ For example, Weaver et al. demonstrated that the AHI was not associated with any changes in non‐PSG measures of OSA severity, whereas the lowest oxyhemoglobin saturation was.^40^ Similarly, Malhotra et al. highlighted that hypoxic burden, defined by the severity and frequency of desaturations, is a crucial factor in predicting the pathophysiological complications of OSA – something the AHI alone struggles at predicting effectively.^41^ Moreover, the existing literature does not show a significant relationship between the AHI and changes in hippocampal volume. For example, a 2020 study by Marchi et al. demonstrated that only mean SpO_2_ was associated with atrophy to cortical and subcortical brain areas, whereas other measurements, such as AHI, were not.^42^ Consequently, in our study, the lack of correlation between AHI and hippocampal volume is likely explained by the fact that AHI does not incorporate desaturations in its definition. Conversely, we saw a decrease in total hippocampal volume in individuals with cognitive impairment due to NVM etiology when assessing the presence of OSA, T90%, and mean SpO_2_ as these metrics incorporated desaturations.

The association between T90% and hippocampal atrophy, as identified in our findings, is not well elucidated in the literature. For example, Marchi et al. showed significant cortical and subcortical atrophy associated with mean O_2_, but not T90%.^42^ Notably, they identified that their dataset was positively skewed, with most participants having a T90% of 0% – a finding that would unlikely yield significant associations. The literature does, however, support T90% as a predictor of hypoxemia. A 2016 study by Baril et al. demonstrated that T90% significantly predicted hypoxemia, while AHI did not.^43^ Given the established knowledge regarding the hippocampus's susceptibility to hypoxic damage, T90%’s predictive ability is conceivable.^4^, ^9^, ^10^, ^11^, ^12^ Thus, while the literature does not directly link T90% to cortical volumes, it highlights its importance in hypoxemia prediction. This aligns with our hypothesis that highlights the importance of desaturations and the associated hypoxemia in the pathophysiology of hippocampal atrophy. Another interesting interpretation is that OSA is only associated with reduced hippocampal volumes when it itself may have secondarily caused neurodegenerative processes for unidentified reasons. The two interpretations are not mutually exclusive and require further investigation.

The association between mean SpO_2_ and reduced hippocampal volumes has been well elucidated in the literature. In the previously discussed 2020 study by Marchi et al., researchers demonstrated that mean SpO_2_ was associated with bilateral hippocampal atrophy.^42^ While this study did not examine a cognitively impaired cohort, their findings demonstrated a measurable hypoxic burden associated with this variable. Consequently, with our findings similarly identifying an association between mean SpO_2_ and reduced hippocampal volumes, specifically within cognitively impaired individuals due to NVM etiology, there is further evidence that this specific form of cognitive impairment leads to increased susceptibility to hypoxic burden.

A secondary analysis examined the effects of OSA, AHI, T90%, and mean SpO_2_ on left and right hippocampal volumes. In individuals with cognitive impairment due to NVM etiology, the hypoxic implications of OSA appeared to be bilateral. While T90% did not show a statistically significant association with reduced right hippocampal volumes, significant associations for OSA, mean SpO_2_, and the T90% for the left hippocampus suggests that a larger sample size could reveal this relationship. While our paper points toward a bilateral effect, further investigation is needed to better understand the left and right hippocampal susceptibility to hypoxic injury.

A subgroup analysis explored associations between sleep variables and hippocampal volumes in NVM cognitive impairment subtypes (NVM). As demonstrated in Table 2, most participants had neurodegenerative diagnoses. Regression models on the neurodegenerative cohort demonstrated that OSA and mean SpO_2_ were linked to reduced hippocampal volumes. The AHI showed no significant association, likely due to its exclusion of desaturations, as discussed previously. Interestingly, T90% was also not significantly associated; however, we hypothesize this was due to the reduced sample size in the subgroup analysis. None of the correlation tests for vascular and mixed subgroups were significant; however, this is likely due to their underpowered nature and limited representation in the cohort (neurodegenerative: *N* = 92; vascular: *N* = 21; mixed: *N* = 12).

Table 1 demonstrates that the age of individuals diagnosed with cognitive impairment of NVM etiology was greater than those with subjective cognitive complaints. This aligns with the existing literature, which identified aging as one of the greatest risk factors for cognitive impairment.^44^, ^45^ Moreover, since the accuracy of diagnostic tools increases as degenerative processes progress over time, this finding is expected. Additionally, we noted that a larger proportion of male participants demonstrated to have OSA. This finding is expected as the increased prevalence of sleep‐disordered breathing in the male population is well established.^46^, ^47^ Interestingly, we observed that a higher proportion of males had cognitive impairments of NVM etiology compared to females. This finding contrasts with the expected demographic trends, which suggest that females typically exhibit a higher prevalence and likelihood of developing cognitive impairment.^48^, ^49^

While our study provides valuable insights into the relationship between OSA and cognitive impairment, its cross‐sectional design limits causal conclusions. Specifically, we cannot definitively ascertain whether OSA and cognitive impairment due to NVM etiology directly cause hippocampal volume loss or if reduced hippocampal volume predisposes individuals to these conditions, though the latter is unlikely. Additionally, sleep data were collected using HSAT or in‐laboratory PSG, a modality often lauded as superior. However, advancements in HSAT protocols have shown both feasibility and equivalency in dementia studies, minimizing the impact of using both methods on our findings.^26^, ^50^, ^51^

Future research should delve deeper into the mechanisms underlying the relationship between OSA, cognitive impairment, and hippocampal atrophy, exploring longitudinal outcomes and treatment efficacy. By elucidating these pathways, we can develop more targeted interventions to improve the cognitive health and quality of life of individuals with cognitive impairments. Additional areas of exploration may involve the assessment of other brain regions, especially when neurodegeneration is not of a hippocampal‐sensitive etiology.

## 5 CONCLUSION

Our study demonstrated that OSA was significantly associated with a decrease in hippocampal volumes, specifically in individuals with cognitive impairment due to NVM etiology. This supports the theory that individuals with cognitive impairment experience underlying neurodegenerative processes, which heighten their susceptibility to the adverse effects of OSA. These findings underscore the importance of considering OSA as a potential modifiable risk factor in vulnerable populations. Recognizing the importance of OSA in this cohort, it is evident that the introduction of both targeted screening and treatment interventions may not only mitigate cognitive decline but also potentially preserve hippocampal integrity.

## AUTHOR CONTRIBUTIONS

M.S.: Conceptualization, study design, data acquisition, data analysis and interpretation, and writing of manuscript. D.T. and I.N.: Interpretation and writing of manuscript. D.R.C., Y.S.C., S.M., M.M., and B.L.: Critical revision of manuscript and data acquisition. A.L., F.C., and M.G.: Conceptualization, study design, and critical revision of manuscript. J.R.: Supervision of data acquisition and critical revision of manuscript. S.E.B.: Conceptualization, study design, supervision of data acquisition, and critical revision of manuscript. M.I.B.: Conceptualization, study design, supervision of data acquisition, analysis and interpretation, and writing of the manuscript.

## CONFLICT OF INTEREST STATEMENT

S.M.: Outside of the submitted work, Dr. Mitchell perceived the following conflicts/disclosures unrelated to this manuscript: Dr. Mitchell receives philanthropic support from the Azrieli Foundation, P. Austin Family Foundation, and Great Gulf Foundation. She has received consulting fees from Eli Lilly Canada, Eisai Canada, and Novo‐Nordisk Canada. M.M.: Outside of the submitted work, Dr. Masellis perceived the following conflicts/disclosures unrelated to this manuscript: Dr. Masellis holds grants to the institution from the Canadian Institutes of Health Research, Washington University, Weston Brain Institute, Women's Brain Health Initiative, and Brain Canada. He has funding for contract research from Roche and Alector. He receives royalties from Henry Stewart Talks. He has received consulting fees from Eli Lilly Canada, Alector, Biogen Canada, Wave Life Sciences, Eisai Canada, and Novo‐Nordisk Canada. He has received speaker honoraria from MINT Memory Clinics, and the ECHO Dementia Series. He has sat on scientific advisory boards for Alzheimer Society Canada and Parkinson Canada; these are unpaid roles. A.L.: Outside of the submitted work, Dr. Andrew Lim reports funding from the Canadian Institutes of Health Research, National Institutes of Health (NIH), and Weston Foundation. F.C.: Research support from the Ontario Ministry of Health and Long‐Term Care Innovation Grant, ResMed Foundation, University Health Network Foundation. M.G.: Outside of the submitted work, Dr. Maged Goubran reports funding from the Canadain Institute of Health Research (CIHR), Natural Sciences and Engineering Research Council of Canada (NSERC), National Institutes of Health (NIH), Canada Foundation for Innovation (CFI), Canada Research Chairs program, Alzheimer's Society, Alzheimer's Association, Digital Research Alliance of Canada, and the Harquail Centre for Neuromodulation. S.E.B.: Outside of the submitted work, Dr. Sandra Black reports funding made to the institution for contract research (no personal investigator fees taken) from Genentech, Optina, Roche, Eli Lilly, Eisa/Biogen Idec, NovoNordisk, Lilly Avid, ICON, Aribio Co. She received peer‐reviewed funding made to the institution (no personal investigator fees taken) from Ontario Brain Institute, CIHR, Leducq Foundation, Heart and Stroke Foundation of Canada, NIH, Alzheimer's Drug Discovery Foundation, Brain Canada, Weston Brain Institute, Canadian Partnership for Stroke Recovery, Canadian Foundation for Innovation, Focused Ultrasound Foundation, Alzheimer's Association US, Queen's University, Compute Canada Resources for Research Groups, CANARIE, Networks of Centres of Excellence of Canada. She received consulting fees and honoraria from Roche, Biogen, NovoNordisk, Eisai Canada, Eli Lilly, and Livagency. She has also participated in advisory boards for the Conference Board of Canada, the World Dementia Council, the National Institute of Neurological Disorders and Stroke, and the Ontario Dementia Care Alliance (ODCA). M.I.B.: Outside of the submitted work, Dr. Mark Boulos reports funding from the Canadian Institutes of Health Research, Heart and Stroke Foundation of Canada, CanStroke Recovery Trials network, Restless Legs Syndrome Foundation, Alternative Funding Plan from the Academic Health Sciences Centres of Ontario, the Temerty Centre for AI Research and Education in Medicine, and the McLaughlin Centre for Molecular Medicine. He also reports consulting fees and honoraria from Jazz Pharmaceuticals, Paladin Labs, Eisai, and the OntarioMD Peer Leader Program; travel support from McGill University; and receipt of sleep equipment or research support from Braebon Medical Corporation, The Mahaffy Family Research Fund, and Green Mountain. M.S., D.T., I.N., D.R.C., Y.S.C., B.L., and J.R.: None. Author disclosures are available in the Supporting Information.

## CONSENT STATEMENT

All participants in the study provided written informed consent for the use of their data.

## Supporting information



Supporting Information

Supporting Information
