# Supplementary material for: Examining the association between sleep apnea and total hippocampal volumes in cognitive impairment
Source: Alzheimers Dement. 2025 Apr 25;21(4):e70183. doi: 10.1002/alz.70183 (PMC12022498; doi:10.1002/alz.70183)
Supplement: Supplementary file 1 — Supporting Information [file ALZ-21-e70183-s002.docx]

**Supplementary Table 1:** Diagnostic criteria for types of cognitive impairment of NVM etiology

| **Disease & Reference** | **Core Diagnostic Criteria** |
| --- | --- |
| **MCI due to AD**  Albert MS, DeKosky ST, Dickson D, et al. The diagnosis of mild cognitive impairment due to Alzheimer’s disease: recommendations from the National Institute on Aging-Alzheimer’s Association workgroups on diagnostic guidelines for Alzheimer’s disease. *Alzheimers Dement*. 2011;7(3):270-279. doi:10.1016/J.JALZ.2011.03.008 | 1. Cognitive concern reflecting a change in cognition reported by patient or informant or clinician (i.e., historical or observed evidence of decline over time) 2. Objective evidence of Impairment in one or more cognitive domains, typically including memory (i.e., formal or bedside testing to establish level of cognitive function in multiple domains) 3. Preservation of independence in functional abilities 4. Not demented 5. Etiology of MCI consistent with AD pathophysiological process (rule out vascular, traumatic, medical causes of cognitive decline, evidence of longitudinal decline in cognition, when feasible, history consistent with AD genetic factors) |

| **Dementia due to AD**  McKhann GM, Knopman DS, Chertkow H, et al. The diagnosis of dementia due to Alzheimer’s disease: recommendations from the National Institute on Aging-Alzheimer’s Association workgroups on diagnostic guidelines for Alzheimer’s disease. *Alzheimers Dement*. 2011;7(3):263-269. doi:10.1016/J.JALZ.2011.03.005 | **All-cause dementia**: MCI criteria and significant interference in the ability to function at work or in usual daily activities  **Probable dementia due to AD** (Meets definition of dementia with following characteristics):   1. Insidious onset. Symptoms have a gradual onset over months to years, not sudden over hours or days; 2. Clear-cut history of worsening of cognition by report or observation; and 3. Amnestic or non-amnestic (language, visuospatial, executive dysfunction) presentation 4. Lack of evidence of alternative etiologies   **Possible:**   1. Atypical course or 2. Etiologically mixed presentation   **Probable/Possible with evidence of AD pathophysiological processes:**   1. Aβ (positive PET or low CSF) 2. Neuronal injury (elevated CSF tau, decreased FDG-PET, disproportionate atrophy of medial, basal, and lateral temporal lobe, and medial parietal cortex on structural MRI) |
| --- | --- |
| **Vascular Cognitive Impairment and Dementia**  Gorelick PB, Scuteri A, Black SE, et al. Vascular contributions to cognitive impairment and dementia: a statement for healthcare professionals from the american heart association/american stroke association. *Stroke*. 2011;42(9):2672-2713. doi:10.1161/STR.0B013E3182299496 | **Probable VaD**  1. There is cognitive impairment and imaging evidence of cerebrovascular disease and  a. There is a clear temporal relationship between a vascular event (eg, clinical stroke) and onset of cognitive deficits, or  b. There is a clear relationship in the severity and pattern of cognitive impairment and the presence of diffuse, subcortical cerebrovascular disease pathology (eg, as in CADASIL).  2. There is no history of gradually progressive cognitive deficits before or after the stroke that suggests the presence of a nonvascular neurodegenerative disorder.  **Possible VaD**  There is cognitive impairment and imaging evidence of cerebrovascular disease but  1. There is no clear relationship (temporal, severity, or cognitive pattern) between the vascular disease (eg, silent infarcts, subcortical small-vessel disease) and the cognitive impairment.  2. There is insufficient information for the diagnosis of VaD (eg, clinical symptoms suggest the presence of vascular disease, but no CT/MRI studies are available).  3. Severity of aphasia precludes proper cognitive assessment. However, patients with documented evidence of normal cognitive function (eg, annual cognitive evaluations) before the clinical event that caused aphasia could be classified as having probable VaD.  4. There is evidence of other neurodegenerative diseases or conditions in addition to cerebrovascular disease that may affect cognition, such as  a. A history of other neurodegenerative disorders (eg, Parkinson disease, progressive supranuclear palsy, dementia with Lewy bodies);  b. The presence of Alzheimer disease biology is confirmed by biomarkers (eg, PET, CSF, amyloid ligands) or genetic studies (eg, PS1 mutation); or  c. A history of active cancer or psychiatric or metabolic disorders that may affect cognitive function.  **Probable VaMCI**  1. There is cognitive impairment and imaging evidence of cerebrovascular disease and  a. There is a clear temporal relationship between a vascular event (eg, clinical stroke) and onset of cognitive deficits, or  b. There is a clear relationship in the severity and pattern of cognitive impairment and the presence of diffuse, subcortical cerebrovascular disease pathology (eg, as in CADASIL).  2. There is no history of gradually progressive cognitive deficits before or after the stroke that suggests the presence of a nonvascular neurodegenerative disorder.  **Possible VaMCI**  There is cognitive impairment and imaging evidence of cerebrovascular disease but  1. There is no clear relationship (temporal, severity, or cognitive pattern) between the vascular disease (eg, silent infarcts, subcortical small-vessel disease) and onset of cognitive deficits.  2. There is insufficient information for the diagnosis of VaMCI (eg, clinical symptoms suggest the presence of vascular disease, but no CT/MRI studies are available).  3. Severity of aphasia precludes proper cognitive assessment. However, patients with documented evidence of normal cognitive function (eg, annual cognitive evaluations) before the clinical event that caused aphasia could be classified as having probable VaMCI.  4. There is evidence of other neurodegenerative diseases or conditions in addition to cerebrovascular disease that may affect cognition, such as  a. A history of other neurodegenerative disorders (eg, Parkinson disease, progressive supranuclear palsy, dementia with Lewy bodies);  b. The presence of Alzheimer disease biology is confirmed by biomarkers (eg, PET, CSF, amyloid ligands) or genetic studies (eg, PS1 mutation); or  c. A history of active cancer or psychiatric or metabolic disorders that may affect cognitive function.  Unstable VaMCI  Subjects with the diagnosis of probable or possible VaMCI whose symptoms revert to normal should be classified as having “unstable VaMCI.” |

| **Dementia with Lewy Bodies**  McKeith IG, Boeve BF, Dickson DW, et al. Diagnosis and management of dementia with Lewy bodies: Fourth consensus report of the DLB Consortium. *Neurology*. 2017;89(1):88-100. doi:10.1212/WNL.0000000000004058 | **Core clinical features (The first 3 typically occur early and may persist throughout the course.)**  Fluctuating cognition with pronounced variations in attention and alertness. Recurrent visual hallucinations that are typically well formed and detailed.  REM sleep behavior disorder, which may precede cognitive decline.  One or more spontaneous cardinal features of parkinsonism: these are bradykinesia (defined as slowness of movement and decrement in amplitude or speed), rest tremor, or rigidity.  **Supportive clinical features**  Severe sensitivity to antipsychotic agents; postural instability; repeated falls; syncope or other transient episodes of unresponsiveness; severe autonomic dysfunction, e.g., constipation, orthostatic hypotension, urinary incontinence; hypersomnia; hyposmia; hallucinations in other modalities; systematized delusions; apathy, anxiety, and depression.  **Indicative biomarkers**  Reduced dopamine transporter uptake in basal ganglia demonstrated by SPECT or PET. Abnormal (low uptake) 123iodine-MIBG myocardial scintigraphy.  Polysomnographic confirmation of REM sleep without atonia.  **Supportive biomarkers**  Relative preservation of medial temporal lobe structures on CT/MRI scan.  Generalized low uptake on SPECT/PET perfusion/metabolism scan with reduced occipital activity the cingulate island sign on FDG-PET imaging.  Prominent posterior slow-wave activity on EEG with periodic fluctuations in the pre-alpha/ theta range.  **Probable DLB can be diagnosed if:**  a. Two or more core clinical features of DLB are present, with or without the presence of indicative biomarkers, or  b. Only one core clinical feature is present, but with one or more indicative biomarkers.  Probable DLB should not be diagnosed on the basis of biomarkers alone.  **Possible DLB can be diagnosed if:**  a. Only one core clinical feature of DLB is present, with no indicative biomarker evidence, or b. One or more indicative biomarkers is present but there are no core clinical features.  **DLB is less likely:**  a. In the presence of any other physical illness or brain disorder including cerebrovascular disease, sufficient to account in part or in total for the clinical picture, although these do not exclude a DLB diagnosis and may serve to indicate mixed or multiple pathologies contributing to the clinical presentation, or  b. If parkinsonian features are the only core clinical feature and appear for the first time at a stage of severe dementia.  DLB should be diagnosed when dementia occurs before or concurrently with parkinsonism. The term Parkinson disease dementia (PDD) should be used to describe dementia that occurs in the context of well-established Parkinson disease. In a practice setting the term that is most appropriate to the clinical situation should be used and generic terms such as Lewy body disease are often helpful. In research studies in which distinction needs to be made between DLB and PDD, the existing 1-year rule between the onset of dementia and parkinsonism continues to be recommended. |
| --- | --- |
